# Giant right coronary artery aneurysm mimicking a right intra-ventricular mass: a case report

**DOI:** 10.1186/s13019-020-1054-0

**Published:** 2020-01-13

**Authors:** Peng Teng, Chengyao Ni, Qianhui Sun, Yiming Ni

**Affiliations:** 1 Department of Cardiothoracic Surgery, The First Affiliated Hospital, College of Medicine, Zhejiang University, #79 Qingchun Road, Hangzhou, Zhejiang People’s Republic of China 310003; 2 Department of Surgical Intensive Care Unit, The First Affiliated Hospital, College of Medicine, Zhejiang University, Hangzhou, People’s Republic of China

**Keywords:** Coronary artery aneurysm, Right coronary artery, Right ventricle, Cardiac mass

## Abstract

**Background:**

Coronary artery aneurysm is a rare condition which constitutes a small proportion of coronary artery disease. Such condition mimicking an intra-cardiac mass is extremely rare and poorly understood.

**Case presentation:**

We present an unusual case of a 53-year-old female with decreased exercise tolerance and lower extremity edema for 3 months. The echocardiography showed moderate tricuspid regurgitation and a right intra-ventricular mass below the tricuspid valve. No ventricular wall akinesia or ST segment change was found on echocardiography or electrocardiogram. Coronary computed tomographic angiography confirmed the diagnosis of intra-ventricular mass with feeding vessel originated from the right coronary artery. The patient was scheduled for tumor resection, and the mass turned out to be a thrombosed giant right coronary artery aneurysm. The patient received successful aneurysm resection and had an uneventful postoperative recovery. Unfortunately, a fistula between right coronary artery and right ventricle was detected on follow-up three months later by echocardiography.

**Conclusions:**

Coronary artery aneurysms presenting as intra-cardiac masses are extremely rare. Comprehensive preoperative evaluation is highly recommended because the surgical strategies for tumor and aneurysm are completely different. Aneurysm resection with bypass surgery is preferred rather than aneurysm repair. To our best knowledge, coronary artery aneurysms presenting as intra-ventricular masses have seldom been reported.

## Introduction

Coronary artery aneurysm is a rare condition of coronary artery disease. It is defined as segmental coronary dilatation that exceeds the diameter of normal adjacent artery or the diameter of the largest artery by 1.5 times [1]. The reported incidence varies from 1.5 to 5% [2], with coronary artery (RCA) more affected [3]. Coronary artery aneurysm is most frequently caused by coronary atherosclerosis while other causes include trauma, autoimmune disease and inflammatory disorders. In some unusual cases, thrombosed coronary artery aneurysms can have clinical manifestation similar to cardiac tumor, such as mass-effect on adjacent structure and cardiac chamber obstruction, which causes great difficulty for diagnosis and treatment. Herein, we report a 53-year-old female presented with a right intra-ventricular mass which was suspected as a malignant cardiac tumor preoperatively and finally diagnosed as a right coronary artery aneurysm. To our best knowledge, less than 10 cases of giant coronary artery aneurysm mimicking as intra-ventricular mass has been published in English over recent decades.

## Case presentation

**Fig. 1:**
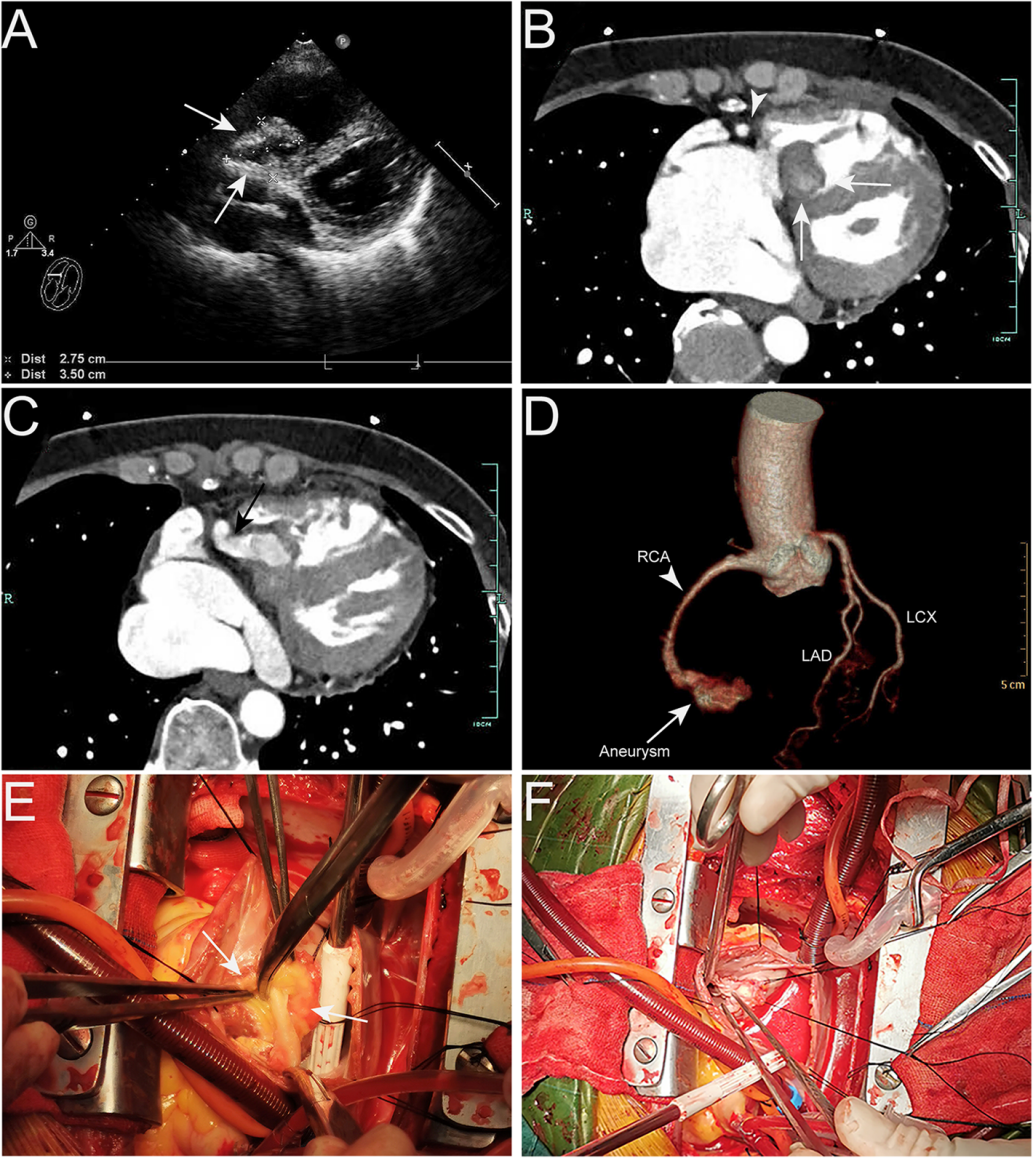
(**A**) TEE revealed a 3.5 × 2.7 cm cystic mass (white arrows) with hyperechoic external capsule locating below the annulus of posterior tricuspid leaflet; (**B, C**) Coronary CTA revealed an irregular mass (white arrows) with internal contrast enhancement and thick external capsule locating below the tricuspid valve. A feeding vessel (black arrow) originating from RCA (white arrow head) was identified; (**D**) 3D reconstruction of coronary arteries confirmed that the mass was supplied by RCA and left coronary artery disease was eliminated; (**E**) After detaching the posterior leaflet from the tricuspid annulus, the mass (white arrows) was detected with collagen-like degenerative fibrous coating; (**F**) The RCA aneurysm was resected and the posterior leaflet was repaired and reattached to the annulus by continuous suture with 5–0 prolene. TEE, transthoracic echocardiography; CTA, computed tomographic angiography; RCA, right coronary artery

A 53-year-old Chinese female with no history of Kawasaki disease or chest trauma was admitted to our hospital because of decreased exercise tolerance and lower extremity edema for 3 months. Physical examination found mild pretibial edema and no heart murmur was heard. Laboratory test revealed elevated eosinophil cell number (0.99 × 10^9^/L) and percentage (14.4%). Tumor markers were negative for malignancy. The transthoracic echocardiography (TTE) showed a cystic mass (3.5 × 2.7 cm) with hyperechoic external capsule locating below the tricuspid annulus (Fig. 1a). Moreover, moderate tricuspid regurgitation was detected, which was mainly attributed to the malcoaptation of leaflets. No ventricular wall akinesia or ST segment change was found on TTE or electrocardiogram. Further assessment with coronary computed tomographic angiography (CTA) showed an irregular mass with internal contrast enhancement locating below the tricuspid annulus (Fig. 1b&c). A feeding vessel originating from RCA was found to supply the mass. No significant coronary stenosis of left anterior descending artery or left circumflex artery was found (Fig. 1d). Considering the mass featuring right-sided location, presence of feeding vessel, irregular shape and internal enhancement, diagnosis of right ventricular malignant tumor was initially made.

Tumor resection was performed via median sternotomy and the cardiopulmonary bypass was established with aortic and bicaval cannulation. After right atriotomy, a mass with about 3.5 × 2.5 cm in size was detected locating at the right ventricle below the tricuspid annulus (Fig. 1e). In addition, the posterior leaflet of the tricuspid valve was extensively affected by the mass. The mass was fully exposed until the posterior leaflet was detached from annulus and was confirmed as a thrombosed right coronary artery aneurysm with collagen-like degenerative fibrous coating. The aneurysm was resected as much as possible and the stump of the feeding vessel was closed by continuous suture with 5–0 prolene. The posterior leaflet was reattached to the annulus by continuous suture with 5–0 prolene (Fig. 1f). Due to absence of evidence of myocardial ischemia either on TTE or electrocardiogram, no bypass surgery was performed.

The histologic and immunohistochemistry study confirmed the diagnosis of coronary artery aneurysm with CD34, CD31 positive and D2–40 negative. The patient had an uneventful recovery and was discharged home on the 7th postoperative day. However, on follow-up three months later, TTE showed a dilated RCA with the proximal diameter of 6.1 mm and a fistula between RCA and right ventricle with a diameter of 2.3 mm. No evidence of remaining or recurrence of aneurysm was detected. The patient had no complaint and no evidence of myocardial ischemia was detected, so we decided to follow up the patient closely. Further treatment will be taken if evidences of myocardial ischemia occur in the future.

## Discussion

Coronary artery aneurysm is defined as segmental coronary dilatation that exceeds the diameter of normal adjacent artery or the diameter of the largest artery by 1.5 times [1]. The reported incidence varies from 1.5 to 5% [2] in autopsy series and 4.9% in the Coronary Artery Surgery Study (CASS) registry [4]. Coronary artery aneurysm is considered to have a predilection for males [5] and RCA [3]. The most common cause is atherosclerosis, which accounts for about 50% of the cases. The other causes are usually involved in the inflammatory processes that affect the arterial wall directly, such as Kawasaki disease, Takayasu’s disease, polyarteritis nodosa, systemic lupus, connective tissue disorders (like Marfan and Ehlers-Danlos syndrome), syphilitic arteritis. Less common causes include congenital coronary artery aneurysm, cardiac lymphoma and iatrogenic trauma during angiography [6].

Coronary artery aneurysms are usually found either as an incidental finding at angiography, or in acute rupture with hemopericardium and tamponade [7], or myocardial infarction due to coronary thrombosis [8]. However, it is extremely rare that coronary artery aneurysms can be misleading as para-cardiac or intra-cardiac masses [9]. In our case, the aneurysm manifested differently since the patient presented with the symptoms of tricuspid incompetence caused by the aneurysm. Preoperative workup disclosed an unexpected right intra-ventricular mass with features of malignancy, including right-sided location, presence of feeding vessel, irregular shape and internal enhancement, which made the initial diagnosis much more difficult.

Echocardiography, coronary CTA and magnetic resonance imaging (MRI) are of great necessity and importance for differential diagnosis between aneurysm and tumor. MRI is the most sensitive modality for characterizing the tumor tissue due to its excellent soft tissue contrast and variable manipulable parameters. In addition, coronary angiography is valuable to confirm diagnosis of coronary artery aneurysm and myocardial infarction, which is helpful for the surgical strategy.

The management of coronary artery aneurysm includes medical intervention, stent insertion and surgical resection [10]. Incidental finding of coronary artery aneurysms could be treated with antiplatelet or anticoagulation agents to prevent potential thrombosis and embolization. Covered stent has also been reported to treat the giant coronary artery aneurysms, which largely depends on the size and location of the aneurysms. Surgery is recommended in the cases with the risk of thrombosis or rupture, especially for cases of giant aneurysms. Surgical resection with bypass surgery is the most frequently chosen and accepted treatment for coronary artery aneurysm [11]. However, the best treatment strategy is still controversial. In our case, we chose aneurysm resection without bypass surgery because the patient presented with no evidence of myocardial ischemia. Unfortunately, such surgical strategy turned out to be not good enough because on follow-up TTE revealed a 2.3 mm-in-diameter fistula between RCA and right ventricle as well as dilated RCA mainly caused by the fistula.

## Conclusion

We experienced a rare case with a giant right coronary artery aneurysm mimicking a right intra-ventricular mass. MRI and coronary angiography are of great importance and value for preoperative diagnosis. We emphasize the necessity of comprehensive evaluation and accurate diagnosis before further treatment. For symptomatic patients with giant coronary artery aneurysms, aneurysm resection with bypass surgery is highly recommended.

## Data Availability

Please contact author for data requests.

## Abbreviations

CTA: Computed tomographic angiography
MRI: Magnetic resonance imaging
RCA: Right coronary artery
TTE: Transthoracic echocardiography
